# On-call medical seclusion reviews: are we meeting MHA code of practice (COP) requirements?

**DOI:** 10.1192/bjo.2021.321

**Published:** 2021-06-18

**Authors:** Oliver Turner, David Leung

**Affiliations:** 1 Leeds and York Partnership NHS Foundation Trust; 2 PICU, Newsam Ward 1, Leeds and York Partnership NHS Foundation Trust

## Abstract

**Aims:**

Are Junior Trainee, Medical Seclusion Reviews complaint with MHA COP Criteria?

**Objectives:**

Are we seeing newly secluded patients on time?

Are we documenting these reviews in clinical notes?

Do documented reviews meet criteria stated by the MHA COP 26.133?

Are we informing Higher Trainees of the need for MDT reviews?

**Background:**

Seclusion is an important aspect of inpatient care. MHA COP Chapter 26 provides guidance for documenting seclusion reviews, ensuring safeguards are in place to protect patient's safety and human rights. Secluded patients require a medical review within 1 hour, and four hourly thereafter, until a higher trainee or Consultant undertake an MDT Review. In our Trust, LYPFT, trainees undertake these reviews. There is noted discrepancy in seclusion review documentation. This audit identifies our compliance with time limits, and whether documentation meets the required criteria in the MHA Code of Practice

**Method:**

Our Sample includes all Out-of-Hour Junior Trainee Medical Seclusion Reviews between 01/01/20 and 01/04/20 at LYPFT. Seclusions were identified from on call logs, and clinical notes were reviewed for a documented seclusion review. The date and time of seclusion are recorded, whether a 1 or 4 hourly review, and the time of review. We recorded any mention of: physical health; mental state; observation levels; recent medication; medication side effects; risk to others; risk to self and the need for ongoing seclusion.

**Result:**

56 episodes of seclusion were identified; all 56 had a documented medical seclusion review. 49 reviews were on time, 4 were late with a documented reason, and 3 were late without. There was documentation of the Higher Trainee being informed in 53 reviews.

No seclusion reviews mentioned all MHA COP criteria. We more frequently mentioned patients’ physical health (51), psychiatric health (52) and need for seclusion (54). 46 seclusion reviews mentioned risk of harm to others; only 3 mentioned risk of self-harm. 25 seclusion reviews mentioned medication, and 5 mentioned review for side effects. 5 seclusion reviews mentioned observation levels.

**Conclusion:**

Our Junior Doctor Seclusion Reviews were not meeting the MHA Code of Practice Criteria, and we believe this to largely be due to lack of awareness of the standards. As such, results have been disseminated to Junior trainees in weekly teaching. We created a medical seclusion review template, adopted by the Trust, to ensure documentation compliance with the MHA COP. Junior doctor inductions now include a presentation regarding Seclusion, the reviews and documentation. We will re-audit in 12 months.

